# A Review of Current Bacterial Resistance to Antibiotics in Food Animals

**DOI:** 10.3389/fmicb.2022.822689

**Published:** 2022-05-12

**Authors:** Chunming Xu, Lingqiang Kong, Hanfang Gao, Xiyu Cheng, Xiumin Wang

**Affiliations:** ^1^School of Light Industry, Beijing Technology and Business University, Beijing, China; ^2^Key Laboratory of Cleaner Production and Integrated Resource Utilization of China National Light Industry, Beijing Technology and Business University, Beijing, China; ^3^College of Life Sciences and Bioengineering, School of Science, Beijing Jiaotong University, Beijing, China; ^4^Key Laboratory of Feed Biotechnology, Ministry of Agriculture and Rural Affairs, Beijing, China; ^5^Feed Research Institute, Chinese Academy of Agricultural Sciences, Beijing, China

**Keywords:** antibiotics, bacterial resistance, food animals, alternatives, strategies

## Abstract

The overuse of antibiotics in food animals has led to the development of bacterial resistance and the widespread of resistant bacteria in the world. Antibiotic-resistant bacteria (ARB) and antibiotic-resistant genes (ARGs) in food animals are currently considered emerging contaminants, which are a serious threat to public health globally. The current situation of ARB and ARGs from food animal farms, manure, and the wastewater was firstly covered in this review. Potential risks to public health were also highlighted, as well as strategies (including novel technologies, alternatives, and administration) to fight against bacterial resistance. This review can provide an avenue for further research, development, and application of novel antibacterial agents to reduce the adverse effects of antibiotic resistance in food animal farms.

## Introduction

Antibiotics have been used extensively in animal feeds for nearly 70 years and approximately 11,000 tons of antibiotics were applied to farm animals in 2019 (National Research Council [NRC], 1980; Food and Drug Administration [FDA], 2019). In Africa, the European Union, and United States, an estimated 50∼80% of all antibiotics are applied to animals, primarily to promote the growth of animals and to prevent bacterial infection (Martin et al., 2015; Van et al., 2020). Antibiotics used in food animals are predicted to increase by 11.5% (up to 200,235 tons) in 2030 (Tiseo et al., 2020). Approximately 75% of antibiotics are not absorbed by the animals and are excreted from the body *via* feces and urine, which can directly contaminate and harm the surrounding environment (Mackie et al., 2006; Zhou et al., 2022). Meanwhile, the misuse or overuse of antibiotics in animal production has led to diverse antibiotic-resistant bacteria (ARB) and antibiotic resistance genes (ARGs), which can be transferred in animals and humans (Martin et al., 2015; Zhang et al., 2015; He et al., 2020; Liu et al., 2022; Yang et al., 2022). There is increasing evidence that antibiotic resistance in humans is mainly related to the wide application of nontherapeutic antibiotics in animals (Martin et al., 2015).

Animal farms are a tremendous reservoir of ARB and ARGs, which is one of the emerging issues globally for human and animal health threats (He et al., 2020; Bai et al., 2021; Wang Y. et al., 2021; Gwenzi et al., 2022; Larsson and Flach, 2022). These ARB and their carried mobile ARGs confer resistance to nine major antibiotic classes, including β-lactams (*bla*), aminoglycosides (*aac*), tetracyclines (*tet*), sulfonamides (*sul*), macrolide-lincosamide-streptogramin B (MLSB; *erm*), FCA (fluoroquinolone, quinolone, florfenicol, chloramphenicol, and amphenicol; *fca*), vancomycin (*van*), colistin (*mcr*), and multidrug (*mdr*), respectively, (He et al., 2020; Larsson and Flach, 2022). Hu et al. (2016b) found that the spread of antibiotic resistance between farm animals and humans can mostly be attributed to the transfer of mobile ARGs, significantly enriched in Proteobacteria (mainly including *Klebsiella pneumoniae* and *Pseudomonas aeruginosa*), Bacteroidetes, Actinobacteria, and Firmicutes. Meanwhile, these shared ARGs between animals and humans confer resistance to *bla*, *aac*, *tet*, chloramphenicol, MLSB, and *sul*; humans shared more ARGs with the chicken than other animals (such as pigs and cattle) from different countries (Hu et al., 2016b).

Various exposure routes of ARB and ARGs originated in farms to humans, including direct or indirect contact with animals, manure or products, and inhalation of bioaerosol that harbors them, may be contributing to increased infections that are hardly treated with antibiotics (He et al., 2020; Bai et al., 2021; Founou et al., 2021; Sirichokchatchawan et al., 2021). Some ARBs (such as *Staphylococcus* spp., *Escherichia coli*, *Salmonella*, etc.) and ARGs can be transferred to humans by direct contact with animals, exposure to animal feces or wastewater, and consumption of contaminated animal food products (including meat, eggs, milk, etc.; Founou et al., 2016, 2021; Khan et al., 2020; Bai et al., 2021; Sazykin et al., 2021). Additionally, airborne ARB and ARGs have also been frequently detected in the farms, and the abundance of some ARGs (such as *tet*, *sul*, *erm*, *bla*, *mec*, etc.) in farm bioaerosols are up to 2.3∼10.6 log copies/m^3^ (Sancheza et al., 2016; Song et al., 2021; Gwenzi et al., 2022). Recent work by Bai et al. (2021) showed that *Staphylococcus*, *Acinetobacter*, and *Sphingomonas* were identified in farm airborne; numerous ARGs (including *mec*, *tet*, *bla*, *aad*, *efr*, *flor*, *sul*, etc.) were dispersed from the animal farms to a distance of 10 km. Multidrug-resistant (MDR) bacteria (including *Staphylococcus* spp., *Enterococcus* spp., *Salmonella* spp., *E. coli*, etc.) and ARGs were transferred to workers and residents in the surrounding environment *via* inhalation, thus, posing the risk of diseases (Bai et al., 2021; Jeżak and Kozajda, 2022; Yang et al., 2022).

**FIGURE 1:**
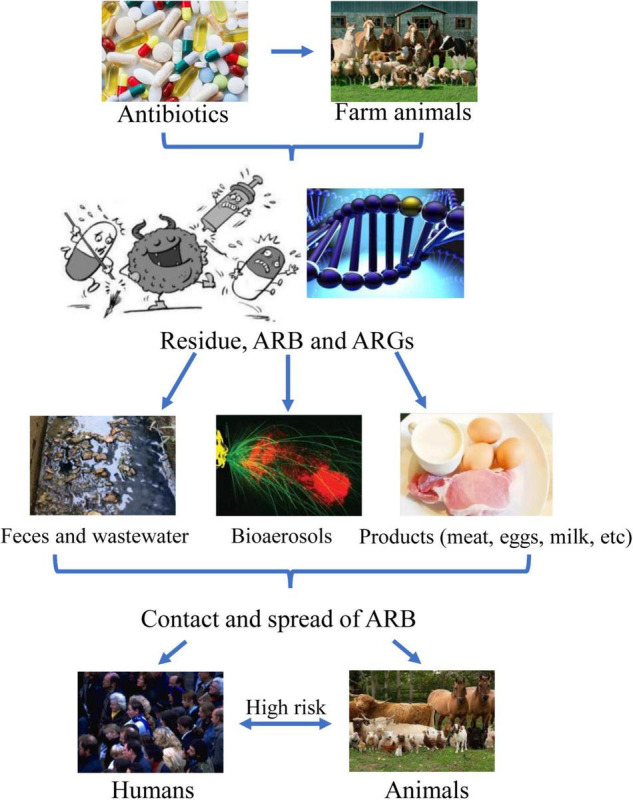
Outlines of antibiotic resistance in food animal farms and risks.

This review introduces a brief outline of the current situations of ARB and ARGs from food animal farms, manure, and wastewater, as well as risks and strategies in the battle against bacterial resistance (Figure 1). By studying current antibiotic resistance in food animal farms, we hope that more people can clearly understand the adverse effects of antibiotic resistance, human exposure, and health risks associated with animal farming activities, which may further help develop more efficient alternatives to antibiotics.

## Bacterial Resistance to Antibiotics in Food Animal Farms

**FIGURE 2:**
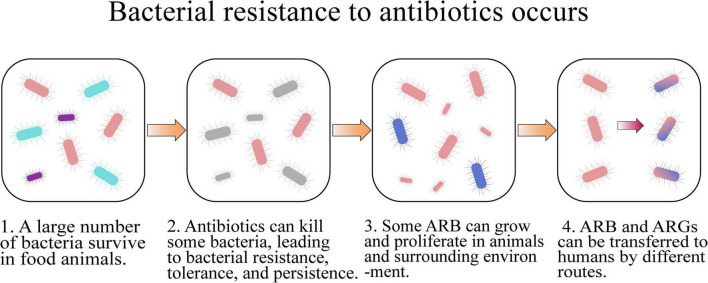
How bacterial resistance to antibiotics occurs.

The routine utilization of antibiotics in food animal farms can accelerate the development of bacterial resistance and dissemination of ARB and ARGs (Figure 2 and Table 1), which is an increasing threat to the health of humans and animals (Van et al., 2020).

**TABLE 1:** Typical antibiotics, antibiotic-resistant genes (ARGs), and antibiotic -resistant bacteria (ARB) in food animal farms.

ARB	Antibiotics	ARGs
Gram-negative bacteria	β-Lactams	*bla*_CTX–M–1_, *bla*_CTX–M–8_, *bla*_CTX–M–14_, *bla*_CTX–M–15_, *bla*_OXA–48_, *bla*_OXA–58_, *bla*_CTX–M_, *bla*_CMY–2_, *bla*_DWA1∼4_, *bla*_DWB1_, *bla*_TEM_, *mec*A, *amp*C, etc.
*E. coli*, *K. pneumoniae*, *P. aeruginosa*, *Salmonella*, *Acinetobacter*, *Sphingomonas*, *Vibrio*, *Aeromonas*, *E. cloacae*, *Clostridium*, etc.	Aminoglycosides	*aac*, *aad*, etc.
	Tetracyclines	*tet*A, *tet*B, *tet*C, *tet*G, *tet*O, *tet*M, *tet*X and *tet*W, etc.
	Sulfonamides	*sul*I, *sul*II, *sul*3, etc.
Gram-positive bacteria	MLSB	*erm*A, *erm*B, etc.
*S. aureus*, *S. intermedius*, *S. hyicus*, *E. faecium*, *E. faecalis*, *E. hirae*, *E. durans*, *E. caaeliflavus*, *E. avium*, *S. agalactiae*, *B. licheniformis*, etc.	FCA	*fca*, *fex*A, etc.
	Vancomycin	*van*
	Colistin	*mcr-1* and *mcr-5.1*

Farming animals are an important source of bacteria with resistance to antibiotics (Founou et al., 2021). A total of 1,739 samples of zoonotic and commensal bacteria were collected from food-producing animals (including cattle, pigs, and chickens) at slaughter in nine European Union countries and their susceptibility to medically important antibiotics was detected by minimum inhibitory concentration (MIC) testing according to Clinical and Laboratory Standards Institute recommendations (de Jong et al., 2018). It was found that *Enterococcus faecium* and *E. faecalis* had higher resistance to tetracycline and erythromycin (27.1∼78.3%) in pigs and chickens than that in cattle (5.2∼30.4%); bacterial resistance to *van* and tigecycline was absent or low; other strains of *E. hirae*, *E. durans*, and *E. caaeliflavus* had no or low resistance to daptomycin, linezolid, tigecycline, and vancomycin. This pan-European Union survey indicated that bacterial clinical resistance to critically important antibiotics commonly used in human medicine was absent or low, except for erythromycin (de Jong et al., 2018). Nesporova et al. (2021) collected 66 samples from 12 poultry farms and analyzed the linkage between antibiotic resistance and hatcheries from Paraguay and Brazil. After whole-genome sequencing, 62 *E. coli* and 22 *K. pneumonia* isolates were identified in these farms. It was observed a higher prevalence of isolates with *mcr-5.1* and *bla*_CTX–M–8_ in three farms, in which chickens were obtained from a Paraguayan hatchery, than others from a Brazilian hatchery; none of the *K. pneumoniae* isolates were related to the Paraguayan hatchery. The results implied that antibiotic resistance may be relevant to the source of the chicken and the farms are considered a reservoir of antibiotic resistance (Nesporova et al., 2021).

Animal gut microbiota is a reservoir of ARB and ARGs, and antibiotic utilization is the main cause of ARG prevalence (Zhu et al., 2020). Zhu et al. collected 30 fecal samples from different bovine farms and explored the prevalence and distribution of ARGs using a metagenomic method. The results showed that the diversity and abundance of ARGs in dairy and beef cattle were similar and significantly higher than those in individual yaks, which may be associated with the long-term use of antibiotics. Additionally, insertions, integrations, and transposons were identified and responsible for the common existence of ARGs, leading to horizontal transfer among microbes, especially pathogenic bacteria (Zhu et al., 2020). Zhang J. et al. (2019) investigated the relationship between animal types and the ARGs emission into the environment in typical swine farms in China and found that approximately 4 ± 1.3 × 10^17^ gene copies of ARGs per day were released from typical swine farms with a stock of 10,000 pigs. Both Firmicutes and Bacteroidetes belong to the dominant phylum in swine manure, but Bacteroidetes, Proteobacteria, and Spirochaetes play a key role in determining the ARGs profiles, indicating that the microbial community in swine manure may mainly contribute to the transfer of ARGs (Zhang J. et al., 2019).

Utilization patterns, excretion, and contamination of 50 antibiotics were investigated in a typical swine farm by Zhou et al. (2013b). A total of 11, 17, and 15 target antibiotics (including tetracycline, bacitracin, chlortetracycline, and florfenicol) were found in feces, flush water, and suspended particles in this farm, which was mainly from the feeds; in comparison, most *sul* (such as oxytetracycline and doxycycline), macrolides, fluoroquinolones, and trimethoprim mainly originated from oral and injection. Moreover, the daily excretion of antibiotics was 1.47∼48.3 mg/d per pig, which is associated with the growth stage of pigs, and chlortetracycline and bacitracin are major contributors. However, simple waste treatments such as anaerobic digesters or lagoons did not effectively remove antibiotics (Zhou et al., 2013b). Novel treatment systems and improved methods are needed for animal wastewater to eliminate veterinary antibiotics.

Bioaerosols from swine confinement buildings (SCBs) are a reservoir of zoonotic pathogens and ARGs from feces, which may cause a high incidence of respiratory infections in pig farmers (Radon et al., 2001; Hoppin et al., 2014; Sigsgaard et al., 2020). Meanwhile, SCB bioaerosols are comprised of various pathogens, such as *Staphylococcus*, *Acinetobacter*, *Pseudomonas*, and *E. avium*, and they can spread over long distances to the external environment, posing a tremendous risk to public health. To determine the bacterial microbiome and resistome profiles of SCB bioaerosols and their correlation with the growth stages of pigs, Yan et al. (2021) collected 24 samples and analyzed the bacterial microbiome and resistome profiles by using the next-generation high-throughput sequencing method. The results showed that the predominant phyla were Proteobacteria and the most abundant ARGs were aminoglycoside resistance genes; bioaerosols from farrowing sows exhibited higher abundances of aminoglycoside, MLSB, bleomycin, multidrug, and trimethoprim resistance genes, but lower abundances of polymyxin and fosmidomycin resistance genes than other stage samples (Yan et al., 2021). It suggested there is a strong relationship between the bacterial microbiome and the resistome in SCB bioaerosols, as well as abundant opportunistic pathogens and potential ARG hosts in the indoor air from pig farms.

Additionally, ARB and ARGs are widespread in aquaculture farms, highlighting a great threat to humans and aquatic organisms (Harnisz et al., 2015; Ranjan and Thatikonda, 2021). The diversity of ARGs in the intestinal bacteria of shrimp was investigated by polymerase chain reaction (PCR) and metagenomic analysis (Liu et al., 2019). Tetracycline, quinolone, sulfadiazine, and erythromycin resistance genes were detected in *Penaeus vannamei* by PCR; 62 different ARGs, which were classified into 21 types (such as *aac*, macrolides, quinolones, *tet*, *bla*, peptide antibiotics, etc.), were identified in the shrimp gut by the plasmid metagenomic method. These ARGs are from *Vibrio* (accounted for 2.8∼51%) and *Aeromonas* (16∼55%) plasmids; especially *Vibrio* may be major bacterial pathogens in shrimp. Notably, the plasmid metagenomic focusing on the mobile genetic elements (MGEs) may have great potential to identify ARGs in complex environments (Liu et al., 2019). Meanwhile, Oviedo-Bolaños collected 450 individuals from tilapia ponds and organ pools in the Northern Pacific region, Costa Rica, and identified ARGs (including *tet*M, *tet*O, *fex*A, and *erm*B) by the PCR method (Oviedo-Bolaños et al., 2021). The results showed that *S. iniae* was not detected, and 60% of the ponds and 46% of the organ pools were positive for *Streptococcus agalactiae* with *tet*O (29.1%), and *tet*M (12.7%) and *erm*B (1.8%), respectively. It is favorable to improve the knowledge of the diseases infected with *Streptococcus* sp. and to efficiently prevent and control these pathogens in tilapia farming worldwide (Oviedo-Bolaños et al., 2021).

Sharma et al. (2020) investigated antibiotic use and animal health management in smallholder dairy farms in four urban and peri-urban areas of India and found that the knowledge level and practices related to antibiotic use and antimicrobial resistance can potentially increase the risk of resistance antibiotic development and its transfer in the community, which is a major global public health threat. Unregulated or inappropriate antibiotic use in animal farms and using different types of antibiotics at the same time are still common practices in developing countries (Gebeyehu et al., 2015; Chowdhury et al., 2021; Geta and Kibret, 2021). In a survey of small-scale farms in Thailand, 57.1% of farmers reported the use of antibiotics for prevention (Lekagul et al., 2020). Some farmers believed that antibiotics could be used to treat all animal diseases, although, 42.9∼72% understood that antibiotic misuse in animals caused bacterial resistance and the spread of ARB (Nuangmek et al., 2018; Ozturk et al., 2019).

## Bacterial Resistance to Antibiotics in Food Animal Manure

Manure, an important fertilizer, contains residual antibiotics, ARB, and diverse ARGs, and its utilization in agricultural fields can affect the structure and function of microflora, leading to an increased abundance of resistant bacteria and resistance genes in the environment (Kim et al., 2010; Wichmann et al., 2014; Van Epps and Blaney, 2016; Xie et al., 2017; Menz et al., 2019).

The vast majority of antibiotics (70%) used in the animals are not absorbed by them and are excreted from their bodies (Mackie et al., 2006). A total of 71 concentrated animal feedlots were collected from Northern China and 24 antibiotics were detected by Li C. et al. (2021). The amounts of antibiotics variated in different animal manures; antibiotic residues in swine manure were 83.18 mg/kg, higher than those of chicken (52.93 mg/kg), beef (37.12 mg/kg), and dairy manures (305 μg/kg), posing serious threats to the terrestrial environment (Li C. et al., 2021). Patyra et al. (2020) detected antibiotics in livestock feces, liquid manure, and digestate from biogas factories located in Poland and Spain by using the liquid chromatography-mass spectrometry (LC-MS) method. The result showed that after administration, active antibiotics were excreted, in some cases 90% of the consumed dose were present in the feces or urine as parent molecules; 18 out of 70 samples were positive for the presence of tetracycline, doxycycline, oxytetracycline, enrofloxacin, chlortetracycline, lincomycin, and tiamulin (Patyra et al., 2020). It suggested that livestock farms may be an important pollution source to transmit antibiotics in feces or manure into the environment. Zhou et al. investigated the excretion masses and environmental occurrence of 50 antibiotics of 11 types in different livestock farms in southern China. A total of 28 antibiotics (such as macrolides, tetracvclines, sulfonamides, fluoroquinolones, bacitracin, lincomycin, etc.) were present in the feeds, wastes, and receiving environments. Antibiotics excreted by swine were mainly from the feeds and 20 times larger than those of dairy cattle, which were from the injection route. The normalized daily excretion masses of chlortetracycline, bacitracin, lincomycin, and tetracycline were 11.6, 3.81, 1.19, and 1.04 mg/day per swine, respectively; chlortetracycline (3.66 mg/day per cattle) contributed to 86% of the antibiotic excretion. Moreover, macrolides, tetracvclines, sulfonamides, fluoroquinolones, etc. were also found in well water, stream, and soil, implying that animal farms may be a vital pollution source of various antibiotics to the receiving environments (Zhou et al., 2013a).

It has demonstrated that a variety of ARB and ARGs in animal manure can confer resistance to *aac*, macrolides, fluoroquinolones, tetracvclines, sulfonamides, phenicols, and glycopeptides, respectively, (Wichmann et al., 2014; Yang F. et al., 2019; Furlan et al., 2020; Huang J. et al., 2021; Li C. et al., 2021). Fournier et al. (2021) collected 81 fecal samples from a pig farm and detected resistance in *Enterobacterales* to colistin, β-lactam, and aminoglycoside. There were 38 β-lactam-resistant *E. coli* isolates and one *Enterobacter cloacae*, which carry plasmids with *bla*_CTX–M–1_, *sul*2, and *tet*A genes, respectively, conferring resistance to β-lactam, *sul*, and tetracycline; it was also identified two colistin-resistant *K. pneumoniae* isolates and a single *E. cloacae* isolate with a truncated *mgr*B, encoding resistance to colistin. The results indicated that a prevalence of β-lactam-resistant *E. coli* can increase the risk of resistance distribution in the environment (Fournier et al., 2021). A total of 40 feces were collected from yaks, beef, and dairy cattle by Wang Y. et al. (2021), and bacterial antibiotic resistance was analyzed by the metagenomic method (Wang W. et al., 2021). It was found that 734 ARG subtypes of 1,688 annotated genes, were associated with β-lactam, tetracyclines, aminoglycosides, and quinolones. Meanwhile, it was found higher abundance of ARGs and a lower level of integron in beef and dairy cattle than in yak, which may be related to the antibiotic selective pressure of their different density feeding patterns. Yaks raised under a low-density feeding pattern may use fewer or no antibiotics, leading to less emergence of resistance. For beef and dairy cattle under high-density feeding patterns, ARGs may be transmitted horizontally from the environment by integron (Wang W. et al., 2021).

Wang X.R. et al. (2021) determined the diversity of ARGs from duck wastes by using the metagenomic method. Among 76 samples, there were 61 variants or novel ARGs, which included five novel β-lactam ARGs (such as *bla*_DWA1∼4_ and *bla*_DWB1_) and two fosfomycin-resistant genes (such as *fosA-like1* and *fosA-like2*). These novel ARGs were related to either hydrolyzation of both penicillins and cephalosporins or MGEs. Noticeably, the MICs of *E. coli* carrying *fosA-like* genes increased by 128-fold, indicating a key resistome reservoir in duck farms and posing a high spread risk (Wang X.R. et al., 2021). Ruuskanen et al. (2016) collected manure samples for storage for 8 months and then investigated the effects of manure on the abundance of resistance genes in the farm environment in southern Finland by PCR and quantitative PCR (qPCR) methods. It was found that the relative abundance of AGRs increased by 4-fold in the soil after manure application; carbapenemase encoding *bla*_OXA–58_ gene was found in four dairy cattle and swine farms, indicating dissemination in the farm environment. In addition, the relative abundance of ARGs in stored manure increased by 5-fold compared with fresh manure roughly, indicating that ARGs are proliferated and dispersed in the farm environment (Ruuskanen et al., 2016).

Antibiotic residues, ARB, and ARGs in manure are considered a growing threat to the human, animal, and environmental health (Lima et al., 2020). Ma et al. (2021) investigated the influence of chlortetracycline on the gut microbiota in pigs and ARGs by 16S rDNA sequencing and metabolomics analysis. The result showed that after the addition of 75 mg/kg of chlortetracycline into the basal diet, the diversity of the gut microbiota in feces and manure dramatically reduced and affected its structure; regulating metabolic pathways mainly involved in the tricarboxylic acid cycle, either propionate or pyruvate metabolism. For 30∼120 day-old pigs, the levels of chlortetracycline residue in feces and the abundance of three tetracycline-resistant genes (including *tet*C, *tet*G, and *tet*W) markedly increased. Additionally, chlortetracycline residues and ARGs abundance in feces and manure gradually decreased with fermentation time, and aerobic composting more potently reduced ARGs abundance (by 84.4%) than anaerobic digestion (79.4%), indicating that fermentation could remove most of the antibiotic residues. The results suggest that although chlortetracycline can reduce the diversity of the gut microbiota in pigs and increase the abundance of ARGs in feces, fermentation may be a common effective method of waste recycling (Ma et al., 2021). Huang J. et al. (2021) investigated the effects of animal manure application on ARG abundance and bacterial distribution in the soil-plant system by qPCR and 16S rRNA sequence analysis. It was found that the utilization of poultry or swine manure remarkably increased ARG abundance in the soil, especially *tet*G and *tet*C; animal manure significantly enhanced ARG abundance in the lettuce endosphere, especially *tet*G in poultry or swine manure and *tet*X in chemical fertilizer, but not in the lettuce phyllosphere. Sphingomonadaceae, Flavobacteriaceae, Comamonadaceae, Flavobacteriaceae, Hyphomicrobiaceae, and others were shared in the soil, lettuce endosphere, and phyllosphere. A significant increase in ARG abundance in the soil-lettuce system caused by animal manure application may be related to the shared bacterial distribution (Huang J. et al., 2021). Therefore, it is crucial to reduce the number of antibiotics and a load of resistant bacteria/genes that end up in the surrounding environment.

## Bacterial Resistance to Antibiotics in Animal Farming Wastewater

Antibiotic-resistant bacteria and ARGs, conferring resistance to antibiotics, have all been found in animal farming wastewater, which is currently considered a serious public health issue (Al Salah et al., 2019; Yang Y. et al., 2019). In recent years, increasing attention is drawn to antibiotic resistance in animal wastewater, especially in developing countries (Van Epps and Blaney, 2016).

Brooks et al. (2014) analyzed the constituents of bacterial pathogens and several ARGs (such as *tet*, *erm*, *mec*, *int*, etc.) in swine manure wastewater by using the qPCR method. Nearly all tested ARGs were found in sow, nursery, and finisher farms; the highest levels of tetracycline- and macrolide-resistant *tet*A and *erm*F genes were approximately 10^9^ and 6 × 10^8^ GU/100 mL in nursery farm lagoon effluent. Other ARGs, including *erm*A, *tet*B, and *int*I, ranged from 10^6^ to 10^7^ GU/100 mL. However, finisher farms had less diversity of microbes and fewer ARGs than others, which may be related to the continuous application of antibiotics in feed. Noticeably, it was found to reduce the methicillin-resistant *mec*A gene as the pigs aged. This result implied that microbial populations and ARGs in swine farm wastewater may be influenced by farm management systems (Brooks et al., 2014).

West et al. (2010) detected bacterial antibiotic resistance and gene transfer in waterways near confined animal feeding operation (CAFO) farms. The results showed that among 830 bacterial isolates, ampicillin-resistant bacteria accounted for 77.1%, and others were resistant to many antibiotics, including kanamycin, ampicillin, streptomycin, oxytetracycline, and chlortetracycline, respectively, which can be affected by CAFO farms. Additionally, 83.3% of the bacterial isolates could transfer resistance genes-*tet*B and *tet*C to *Salmonell*a *enterica* Typhimurium by conjugation, indicating a high risk for pollution of MDR bacteria from animal waste (West et al., 2010).

Al Salah et al. (2019) detected the bacterial abundance and ARGs in Sub-Saharan rivers receiving animal farming wastewaters in Kinshasa, Congo by the qPCR method. It was found that the two rivers are a reservoir of resistant bacteria (such as *E. coli*, *Pseudomonas*, and *Enterococcus*), and β-lactam, sulfonamide, and tetracycline resistance genes (including *bla*_OXA–48_, *bla*_CTX–M_, *sul*1, *sul*2, *sul*3, and *tet*B, respectively); both *sul*1and *sul*2 genes were the most abundant and the most detected ARGs. Contamination sources mainly originated from pigs and anthropogenic activities, and animal farming wastewaters did not exclusively lead to antibiotic resistance profiles. This serves as a very valuable tool to evaluate antibiotic resistance contaminants in aquatic ecosystems (Al Salah et al., 2019).

Zhao et al. (2022) identified ARGs in decentralized sewage treatment facilities from both livestock and households in developed rural areas in eastern China using metagenomics. A total of 825 bacterial-related and 19 bacteriophage-related ARGs in the rural wastewater were identified; the dominant ARGs were resistant to bacitracin, indicating the difference between rural and municipal wastewater. Bacteriophage ARGs (including *gyr*A, *drf*E, *rpo*B, and *par*C) mainly confer resistance to rifampin, trimethoprim, aminocoumarin, lipopeptide, fluoroquinolone, and pyrazinamide. *Acidovorax* and *Prymnesiovirus* are the dominant hosts of ARGs. Noticeably, bacteriophages, as a reservoir of ARGs, are also involved in the dissemination of ARGs (Zhao et al., 2022). Therefore, we should pay attention to the transmission of ARGs by bacteria and phages in wastewater treatment facilities in the future.

Antibiotic residues and ARGs in farm wastewater can dramatically destroy the micro-ecological balance and enhance the prevalence of resistant bacteria in the environment, which urgently need environmental stewardship in some farm operations (Van Epps and Blaney, 2016; Goulas et al., 2018).

## Potential Risks of Antibiotic Resistance in Food Animal Farms

**FIGURE 3:**
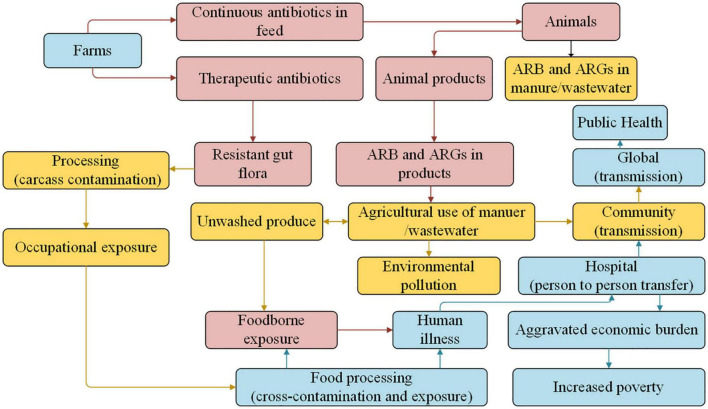
Multiple pathways involved in ARB and ARGs in the food animal and human health (Koch et al., 2017).

Overuse or misuse of antibiotics to treat infectious diseases in animals can lead to the residues of antibiotics and ARGs, which is an increasingly serious threat to human health worldwide and food security (Martin et al., 2015; Founou et al., 2021). As shown in Figure 3, ARB is transferred to humans by direct contact with farm animals, through exposure to animal manure, wastewater, or aerosol, and by consumption of uncooked animal products (such as meat, eggs, milk, etc.; Li et al., 2016; Koch et al., 2017; Deng et al., 2018; Li C. et al., 2021; Liao et al., 2021).

### Direct Contact With Animals

Several studies have demonstrated that farm animals are a reservoir of ARB (such as *E. coli*, *K. pneumonia*, *Salmonella*, *S. aureus*, etc.) and ARGs (Table 1), and people can be infected with ARB through close contact with farm animals (Tomley and Shirley, 2009; Dierikx et al., 2013; McDaniel et al., 2014; Saliu et al., 2017; Founou et al., 2021; Meijs et al., 2021).

The occurrence and prevalence of extended-spectrum β-lactamase (ESBL)- and AmpC β-lactamase (AmpC)-producing *E. coli* and *K. pneumoniae* in farmworkers and household members in Netherlands were investigated by Meijs et al. (2021). PCR and sequencing results showed that ESBL-producing *E. coli* and *K. pneumoniae* in workers accounted for 9.8%, higher than that in the Dutch population (5%), which may be related to direct contact with animals. The resistant genes*-bla*_CTX–M–15_ (48.5∼64.3%), *bla*_CTX–M–14_ (7.1∼18.2%), and *bla*_DHA–1_ (12.1%) were the most prevalent ESBL ones in workers. Additionally, 17.4% of household members had ESBL-producing *E. coli* and *K. pneumonia* and 13% of members carried the same ESBL gene as workers. The data indicated that direct or indirect contact with animals is a potential source of ARB and ARGs (Meijs et al., 2021).

Huijbers et al. (2014) investigated whether resistant *E. coli* can be transferred from broilers to farmers. The result showed that the prevalence of ESBL- and AmpC-producing *E. coli* among people on farms was up to 14.3∼27.1% after contact with infectious live broilers, higher than those among partners and family members who are not living on farms (11.4∼15.7%). This result was consistent with that of previous studies, in which direct contact with broilers may have a high risk for ESBL- and AmpC-producing *E. coli* among humans (Huijbers et al., 2013, 2014). Subsequently, Huijbers et al. (2015) evaluated the prevalence of methicillin-resistant *S. aureus* (MRSA) and AmpC-producing *E. coli* among broilers at different ages and workers. No MRSA isolates were found in broilers and workers; the prevalence of ESBL- and AmpC-producing *E. coli* in farms was 87.5 (34 day-age) and 100% (68 day-age), higher than that of workers (18.5%). Meanwhile, both *bla*_CTX–M–1_ and *bla*_CMY–2_ resistant genes were detected in broilers and workers, which may be associated with contact with live animals. This result stated that direct contact with animals is a high-risk factor for the carriage of ARB and ARGs (Huijbers et al., 2015).

Vines et al. (2021) collected 210 fecal samples from farm animals and the farmer and analyzed the spread of *mcr*-associated with plasmids among *E. coli* by multiplex PCR. It was identified 18 colistin-resistant *E. coli* isolates carrying *mcr*-1 (including 13 from calves, four from pigs, and one from the farmer, respectively) and 33 virulence factors. Moreover, the *mcr*-1 gene was transferred from the calf to the farmer, posing the risk of a farm as a reservoir of ARB with zoonotic potential (Vines et al., 2021).

Workers on farms may have an increased risk of acquiring ARB and ARGs due to their direct contact with animals and transferring between persons, except for exposure to antibiotics in their daily practice environment (Meijs et al., 2021).

### Exposure to Animal Manure, Wastewater, or Bioaerosols

Animal manure and wastewater have been regarded as the hotspots for the transmission of ARB and ARG from animals to humans (Sirichokchatchawan et al., 2021). A large amount of evidence has demonstrated that animal waste and slaughterhouses are a revisor of MDR bacteria, indicating an occupational risk to workers (Aworh et al., 2021).

Aworh et al. (2021) analyzed the prevalence of fecal carriage of MDR *E. coli* among 118 healthy workers in a slaughterhouse by using the Kirby-Bauer disk diffusion method. It was found that the prevalence of MDR *E. coli* among butchers was up to 50%, higher than that among cleaners at the slaughterhouse. This result may be related to keeping animals at home and eating or collecting waste at the slaughterhouse (Aworh et al., 2021). These data provide further evidence demonstrating the occupational hazards for people working in slaughterhouses.

Farm bioaerosols generated during farming practices have also been regarded as a reservoir of ARB and ARGs and may pose a high risk to animal and human health (McEachran et al., 2015; Van Leuken et al., 2016; Anand et al., 2021; Gwenzi et al., 2022). Bai et al. (2021) investigated the spread of ARB from animal bioaerosols and found that ARB and ARGs in bioaerosols could easily be transmitted from animal farms to the environmental dust through the natural wind. Guo et al. analyzed the abundance and transmission route of airborne ARGs in pig farms (Song et al., 2021). The result showed that both *Clostridium* and *Streptococcus* were dominant ARBs, and the abundance of ARGs and MGEs in bioaerosols during winter was higher than that during the summer. The concentration of dominant *tet*M in bioaerosols was 6.3 log copies/m^3^, higher than that in feces (10.6 copies/m^3^), and the transposon *IS*613 had the highest abundance of 4.9 log copies/m^3^. Additionally, the wind speed and high temperature could increase the spread of ARGs in bioaerosols. Noticeably, fecal contribution to ARB in bioaerosols accounted for 59.4% in winter, higher than that in summer (19.9%). Horizontal gene transfer of ARGs in bioaerosol exhibited a higher possibility in winter (77.8%) than that in summer (12.0%; Song et al., 2021). These ARB and ARGs in bioaerosols can be inhaled into the respiratory tract of animals and humans, causing negative health effects such as allergies or asthma (Gao et al., 2018).

Therefore, frequent exposure to animal manure, waste, or bioaerosol, which contain a large number of ARB and ARGs, is considered one of the potential human health threats.

### Consumption of Food Animal Products

The threat of antibiotic over-usage in food-production animals and the associated emergence of antibiotic resistance in zoonotic bacterial pathogens have been recognized for decades (Overdevest et al., 2011). Animal products including meat, eggs, and milk have been proven to be a key route of extraintestinal MDR pathogens, especially for *E. coli* and *Salmonella*, posing a potential health risk to consumers (Borzi et al., 2018; Yamaji et al., 2018; Diaz-Jimenez et al., 2020). Previous studies have demonstrated the contamination rate of animal meat carried ARB reached up to 100% (Geser and Hächler, 2012; Saliu et al., 2017).

The prevalence of ESBL genes of *Enterobacteriaceae* and their relationship between animal meat and humans in Netherlands were determined by Overdevest et al. (2011). The prevalence of ESBL genes in chicken, mixed meat, beef, and pork was 79.8, 9.1, 4.7, and 1.8%, respectively, indicating higher ESBL contamination of chicken meat. Moreover, 6.7% of patients have been infected with ESBL-producing *E. coli* and 4.9% of samples carried ESBL genes, such as *bla*_CTX–M–15_, *bla*_CTX–M–1_, and *bla*_CTX–M–14_ (Overdevest et al., 2011). Deng et al. analyzed 152 *Salmonella* isolates from retail foods of animals by susceptibility testing and found that 92.8% of bacteria were resistant to over one antibiotic. A higher resistance to oxytetracycline and trimethoprim (80.9∼64.5%) was also observed than other antibiotics (28.9∼10.5%), including amoxicillin, ampicillin, levofloxacin, ciprofloxacin, and gentamicin, respectively, while both *bla*_TEM_ and *tet*A genes occupied 44.7%. Additionally, *tet* and *sul* genes were closely related to disinfectant or heavy metal resistance genes (such as *pco*C, *pco*R, or *qac*). This result indicated that retail meat may be a reservoir for the transmission of antibiotic-resistant *Salmonella* (Deng et al., 2018), becoming a serious health threat to humans.

Zou et al. (2021) evaluated the prevalence of extraintestinal pathogenic *E. coli* (ExPEC) in healthy chickens. Among 926 *E. coli* isolates, there were 22 ExPEC ones (accounting for 2.4%), and most of them carried both *bla*_CTX–M_ and *fos*A3 resistance genes, conferring resistance to β-lactam and fosfomycin, respectively. *E. coli* O78 was found to be the most predominant one of six serogroups, which commonly occurred in humans. It suggested that these ExPEC may be transmitted to humans by food supply (Zou et al., 2021).

Cornejo et al. (2020) collected chicken eggs from 83 backyard poultry production systems (BPS) in central Chile and detected antibiotic residues in eggs by using a Four-Plate Test screening assay. The results showed that eggs from 61, 53, and one BPS were positive for at least one antibiotic, more than one, and all four types of antibiotics; the positivity of BPS to *bla*, *aac*, *tet*, and macrolides was up to 59, 56.6, 20.5, and 13.3%, respectively. Antibiotic residues in eggs provide supporting information for non-addressed food safety and animal management, highlighting a potential health risk to consumers (Cornejo et al., 2020).

Balemi et al. (2021) detected the antibiotic resistance of 564 bacterial isolates from California Mastitis Test positive milk from dairy cows, camels, and goats against antibiotics commonly used in Southern Ethiopia. The results showed that coagulase-negative *Staphylococcus* species (accounted for 39.1%), *S. aureus* (17.2%), *S. hyicus* (14.1%), *S. intermedius* (9.4%), and *E. coli* (9.4%) were major pathogens; *E. coli* isolates from both cows and goats were resistant to vancomycin, ceftriaxone, spectinomycin, and doxycycline, and they were intrinsically resistant to penicillin; *S. aureus* isolates from all animals were resistant to spectinomycin, penicillin G, and clindamycin, indicating a public health risk of infection with MDR bacteria due to regular consumption of raw milk (Balemi et al., 2021).

These findings suggest that the food chain of animal products has been one of the important sources of ARB and ARGs transmission between animals and humans, posing a serious global public health threat. The abundant presence of ARB and ARGs transferring from animals to humans may have profound influences on future treatment, highlighting the importance of implementing hygiene measures to decrease the spread risk, thus, it urgently needs to be integrated, multi-sectoral, and global strategies (Overdevest et al., 2011; Stefanska et al., 2021).

## Strategies to Reduce Antibiotic Resistance From Food Animal Farms

Vast amounts of antibiotics are introduced into animal farming and cause the growing prevalence of ARB and ARGs (Table 1), which is an increasing public health concern (Founou et al., 2021; Zalewska et al., 2021). More attention should be given to the antibiotic residues in animal waste to prevent its transfer to the environment, and effective strategies are required to minimize bacterial resistance in animal farms.

### Novel Technologies

**FIGURE 4:**
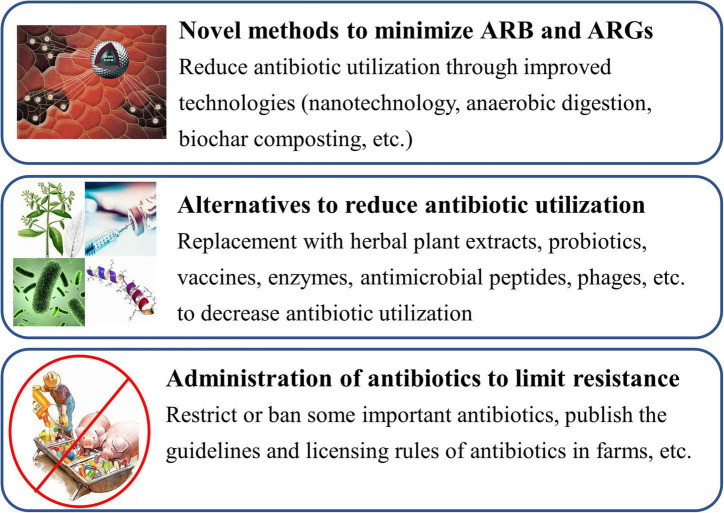
Strategies to minimize the antibiotic resistance in food animal farms.

A few different methods, such as nanotechnology, anaerobic digestion, biochar composting, etc., have been developed to minimize ARB and ARGs in recent years (Figure 4; Yu et al., 2017; Goulas et al., 2018). Yu et al. (2017) collected water samples (including feedlots, fishponds, and wastewater treatment plants) and performed the removal test of ARGs for water by graphene oxide (GO) nanosheet. The results showed that GO nanosheet could nonspecifically bind to the ARGs (such as *tet*A, *erm*B, *amp*C, and *sul*2) by π-stacking interactions, and approximately 80% of ARGs can be removed from water by GO. The removal efficacy of GO nanosheet for ARGs reduced by <40% after 5 regeneration cycles, indicating excellent stability and reusability of GO nanosheet. GO nanosheet may be a desirable candidate for the efficient treatment of ARGs in animal wastewater or other water (Yu et al., 2017). Visca et al. (2021) firstly performed the effects of anaerobic digestion on the removal of ciprofloxacin, sulfamethoxazole, and enrofloxacin and their ARGs in a dairy farm. The result showed that degradation of sulfamethoxazole, ciprofloxacin, and enrofloxacin was 100, 92, and 84%, respectively; removal efficiency of their resistance genes was 78.3% (ciprofloxacin), 50.3% (enrofloxacin), and 37% (sulfamethoxazole), respectively. It noted that anaerobic digestion can be a promising practice for minimizing antibiotic residues and ARGs in animal waste (Visca et al., 2021). Additionally, both tetracycline and sulfadimethoxine were added into dairy manure and treated for 44 days; the effects of anaerobic digestion on the removal of antibiotics and ARGs were firstly investigated by Schueler et al. (2021). The results showed that tetracycline and sulfadimethoxine were reduced by 0∼96% and >99%, respectively, during the digestion processing, and were accompanied by a decline in *tet*M gene copies. However, the reduction in sulfadimethoxine was uncorrelated with a decline in *sul*1 gene copies. Additionally, after the addition of antibiotics at the concentration of 10 mg/L, methane production decreased by 7.8% when compared with the manure-only reactors (Schueler et al., 2021). It indicated that anaerobic digestion may offer the opportunity to remove some antibiotics and reduce antibiotic resistance in ecosystems.

Tao et al. (2014) determined ARGs in wastewater treatment plants of six large swine farms in Taiwan by qPCR. All resistant genes (including *tet*W, *tet*A, *sul*I, *sul*II, and *bla*_TEM_) were detected in six livestock farms, indicating that resistance to tetracyclines, sulfonamide, and β-lactams is prevalent in the wastewater treatment of livestock farming. The highest levels of five ARGs were found in the anaerobic treatment tank and the lowest in the activated sludge unit and the effluents. The removal efficiency of these ARGs ranged from 33.30 to 97.56% in influx and efflux samples. It suggested that anaerobic treatment of livestock farming wastewater may reduce the dissemination of ARB (Tao et al., 2014).

Liao et al. (2021) explored the effects of composting storage (4 weeks) on the abundance of residual ARGs and MGEs by using metagenomics, qPCR, and direct culturing methods. It was observed that 43.8% of ARGs and 39.9% of MEGs rapidly rebound during the 1st week of the composting phase, which was mainly caused by the regrowth of indigenous ARB; residual ARGs and MGEs also rebound at the end of the storage, which was associated with external airborne bacterial transmission (Liao et al., 2021). Moreover, hyperthermophilic composting with a relatively higher temperature (up to 90^°^C) was more efficient at reducing ARGs, MGEs, and their associated bacteria than that of conventional composting (Liao et al., 2018, 2021), indicating the importance of exploring more efficient compost strategies to inhibit the rebound of ARGs and MGEs. Zhang M. et al. (2019) detected the effects of manure composting on eliminating 41 veterinary antibiotics and found that 64.7% of antibiotics were removed after the 171 day-composting. The removal rates for macrolides, *tet*, and *sul* were 100, 73.4, and 45.1%, respectively, except for fluoroquinolones (negative), indicating their different removal rates. This may be associated with the compost’s physico-chemical parameters, such as temperature, moisture, and the ratio of total organic carbon and Kjeldahl nitrogen. During composting, low moisture and pile turning frequency could inhibit further dissipation of antibiotics (Zhang M. et al., 2019). This result is consistent with previous studies in which low moisture and high temperature could cause early dehydration and degradation (McCartney and Tingley, 1998; Ho et al., 2013; Mitchell et al., 2015). Utilization of compost manure with antibiotic residues can cause soil contamination, posing a risk of antibiotic resistance selection to the soil ecosystem (Zhang M. et al., 2019). Liu et al. (2021) explored the influences of co-composting on the removal of antibiotics and ARGs in pig manure by using different biomass ratios of microbial agents, including *Phanerochaete chrysosporium* (p), *Bacillus licheniformis* (b), and *Aspergillus niger* (a), respectively. There were four composting piles of A (p:b:a = 1:0:0), B (control), D (p:b:a = 1:5:5), and G (p:b:a = 1:4:0), respectively. The best removal effect of tetracycline (93.7%) and oxytetracycline (87.8%) was found in composting pile D, higher than that in pile B (75.9 and 58.6%, respectively); the highest removal of doxycycline (98.6%) and enrofloxacin (89%) existed in pile A, higher than that in pile B (64.2 and 65.4%, respectively). This result is superior to a previous study in which the removal rate of antibiotics ranged from 28.8 to 77.8% (Lu and Lu, 2019). Moreover, ARGs in pile D, except those for *sul*, were reduced by 1.059 × 10^–3^∼10^–2^ gene copies/16S rRNA copies compared with those in pile B. In pile A, the relative abundance of integrase genes-*int*I1 and *int*I2 effectively decreased (Liu et al., 2021).

Zhang et al. (2020) detected variations in AGRs of feces of the dairy cow during dairy manure piled up by metagenomic analysis. The results showed that the diversity of pathogens and abundance of ARGs had a significant increase in accumulated manure. Meanwhile, bacterial pathogens from manure were transferred to the environment when manure was applied as fertilizer (Zhang et al., 2020). Thus, effective management and proper measures should be taken in compost piles and manure application to reduce the dissemination of antibiotics in the environment.

Interestingly, Tian et al. (2021) collected 60 samples from 15 earthworm farms and investigated the fate of ARGs during earthworm conversion of cow dung in northern China. It was observed that the abundance of some ARGs sharply declined after composting with earthworms, but some ARGs remained in earthworms and vermicompost, with the level of 10^–1^ to 10^–2^ copies/16S copies. In 15 farms, the most abundant tetracycline-resistant *tet* gene was up to 10^–6^∼10^–1^ copies/16S copies and other high-risk β-lactam resistance *bla* genes were also prevalent, indicating the dispersion risk of ARGs during the earthworm conversion of cow dung. Additionally, other factors, such as heavy metals and total nitrogen content, may be linked to the abundance of some ARGs (Tian et al., 2021).

Liu et al. (2013) investigated the removal capability of some antibiotics (such as ciprofloxacin HCl, oxytetracycline HCl, and sulfamethazine) and ARGs (including *tet*O, *tet*M, and *tet*W) from swine wastewater by using volcanic- and zeolite-medium system treatment. The qPCR results showed that antibiotic concentrations significantly decreased in two vertical flow constructed wetlands, and oxytetracycline HCl had the highest removal rate, superior to ciprofloxacin HCl and sulfamethazine. The absolute abundance of *tet*O, *tet*M, and *tet*W declined by 50%, as well as one order of magnitude in volcanic and zeolite systems. Meanwhile, the zeolite-medium system more efficiently eliminated antibiotics from wastewater than the volcanic-medium one, which may be related to different pH and average pore sizes of media. More antibiotics were adsorbed in the soil, higher than that in the media and vegetation. This implied that soil is responsible for antibiotic removal from wastewater in constructed wetlands, which can adsorb all almost antibiotics (>90%) from the aqueous phase (Liu et al., 2013).

Except for the above, other methods (such as photocatalyst, biochar composting, nanotechnology, reactors, etc.) used in diverse environmental samples can be potentially applied for the degradation of antibiotics and ARGs in animal farms due to excellent stability and recyclability (Tables 2, 3; Chen et al., 2018; Peng et al., 2018; Shi et al., 2018; Srinivasa Raghavan et al., 2018; Guo et al., 2020; Hu et al., 2020; Meng et al., 2020, 2021; Huang B. et al., 2021; Li S. et al., 2021; Wang J. et al., 2021; Zhou et al., 2021).

**TABLE 2:** Other novel methods to reduce ARB and ARGs.

Methods	Characteristics	Samples	References
Fe_2_O_3_ nanoparticles	Efficient and rapid degradation of organic pollutants in water	Antibiotic water	Guo et al., 2020
Photocatalys	Excellent stability and recyclability	Antibiotic wastewater	Zhou et al., 2021
Biochar composting	Effectively prevent greenhouse gas emissions and avoid environmental pollution	Mature compost	Huang B. et al., 2021
Bimetallic nitrogen-doped porous carbon	Renewable energy production and wastewater treatment	Antibiotic wastewater	Li S. et al., 2021
Moving bed biofilm reactor (MBBR) system	Efficient process for treating wastewater with poor biodegradability	Antibiotic wastewater	Peng et al., 2018
Microalgal pretreatment	Effective removal of antibiotic residues and achievement of organics	Saline antibiotic wastewater	Shi et al., 2018

**TABLE 3:** Diverse reactors to treat antibiotic wastewater.

Methods	Characteristics	Sources	References
Anaerobic membrane bioreactor (AnMBR)	Low energy consumption	Artificially formulated antibiotic wastewater	Chen et al., 2018
Up-flow anaerobic bio-electrochemical system (UBES)	Higher removal performance	Antibiotic wastewater from park and pharmaceutical company	Hu et al., 2020
Expanded granular sludge bed (EGSB) reactor	Low cost, low sludge production and long-term stable operation	Artificially formulated antibiotic wastewater	Meng et al., 2020, 2021
Osmotic membrane bioreactor (OMBR)	High rejection by the forward osmosis membrane, water reclamation and facilitated nutrients recovery	Synthetic wastewater	Raghavan et al., 2018
Up-flow blanket filter (UBF) reactor	Economical, exhibited favorable performance	Simulated antibiotic wastewater	Wang J. et al., 2021
Anoxic/oxic-membrane bioreactor (A/O-MBR)	High removal efficiency of bulk pollutants (such as phosphorus, ARGs and antibiotics)	Synthetic wastewater	Wen et al., 2018

### Development of Alternatives to Antibiotics

Some effective or commercially viable alternatives have been implemented by animal farmers or are under development; various alternatives (such as herbal plant extracts, probiotics, vaccines, enzymes, antimicrobial peptides, phages, etc.) to antibiotics are available to reduce antibiotic use in food animals in farms (Figure 4; Cheng et al., 2014; Callaway et al., 2021; Sirichokchatchawan et al., 2021). Among them, some herbal plant extracts (such as tannins, saponins, essential oils, etc.) and probiotics are regarded as powerful natural alternatives to antibiotics due to their various functions including antimicrobial activity, antioxidant activity, anti-inflammatory, immunomodulation, etc. (Huang et al., 2018; Farha et al., 2020; Hernandez-Gonzalez et al., 2021).

Tannins are a group of polyphenolic compounds with a molecular weight of 0.5∼30 kDa that are widely distributed in plants and have potent antibacterial activities against *E. coli*, MRSA, *Shigella flexneri*, and others (Si Heung et al., 2012; Huang et al., 2018; Girard and Bee, 2020). Similarly, plant saponins are comprised of sugar (glycon) and nonsugar ones (aglycon) connected by a glyosidic linkage and exhibit excellent activity against *Salmonella*, *E. coli*, and *Streptococcus aureus in vitro* and chickens (Khan et al., 2018). Garlic oils have long been known as the most effective plant extract to treat bacterial infections (Navidshad et al., 2018). Magrys et al. (2021) investigated the antibacterial activity of garlic extracts against *Enterococcus faecalis*, *S. aureus*, *P. aeruginosa*, and *E. coli*. It was found that garlic extracts had potent bactericidal activity against resistant *S. aureus*, *E. coli*, *P. aeruginosa*, and *K. pneumonia*; garlic extracts showed combinations with gentamycin and ciprofloxacin (Magrys et al., 2021). These functional secondary metabolites used in animals can improve gut health and digestive performance to animals, indicating their alternative treatment to improve the health of animals (Redondo et al., 2014).

Additionally, some probiotics used in animal production have increased significantly over 10 years. Particularly, both *Bacillus* and lactic acid bacteria (LAB) are considered effective and safe alternatives to antibiotics for animal production due to their high stability *in vivo* (Dowarah et al., 2017; Mingmongkolchai and Panbangred, 2018). *Bacillus* belongs to Gram-positive bacteria and can form spores, which are favorable for long-term storage; *Bacillus* can improve growth performance, immunity function, and gut health in animals (Grant et al., 2018; Hu et al., 2018; Ramlucken et al., 2020). LAB is a class of bacteria that produce bacteriocins and can prevent bacterial resistance, so LAB is a powerful alternative to antibiotics (Hernandez-Gonzalez et al., 2021). Musliu et al. (2021) investigated the antibiotic potential of LAB extract and supernatant on MDR pathogenic bacteria from animal farms. The result showed that both crude culture extract (CCE) and cell-free supernatant (CFS) of LAB exhibited significant inhibition zones (7∼25 mm) against *E. coli*, *S. aureus*, and *S. agalactiae*, respectively. Meanwhile, CFS of LAB was more efficient than CCE, which can contribute to declining the burden of infection caused by ARB in farms (Musliu et al., 2021).

The crude extract isolation of herbal plants and the cultivation of probiotics is a simple process that does not require the use of any sophisticated equipment and techniques, thus they may be recommended as ones of the most promising alternatives to antibiotics and used in the treatment of MDR pathogens in both local and modern animal farms to reduce antibiotic usage (Dowarah et al., 2017; Huang et al., 2018; Abdallah et al., 2019).

### Stringent Administration of Antibiotic Application

To minimize antibiotic resistance, a reduction or limitation of antibiotics used in food animals is likewise a very important strategy (Lee et al., 2013). Firstly, many antibiotics cannot be used in food animals as growth promoters and prevention purposes. The European Union, the United States, China, and other countries have banned or restricted the use of some antibiotics as growth promoters and disease prevention in food animals (Table 4); however, use in healthy herds for disease prevention was not prohibited (Food and Drug Administration [FDA], 2017; Salim et al., 2018; Heffernan, 2022). Antibiotics are used only in food animals to treat clinical diseases in accordance with the instructions for use. Meanwhile, government officers should restrict or ban some important antibiotics such as colistin and *van*, which are last-hope or last-resort ones (Figure 4; Qiao et al., 2018). Early in 2012 and 2016, the plasmid-mediated *van*-resistance enterococci and colistin-resistance *E. coli* were found in broilers, pigs, pork products, and humans in Sweden and China, respectively, (Nilsson, 2012; Hu et al., 2016a; Qiao et al., 2018). Colistin-resistance gene (*mcr*) existed in more than 11 bacterial species and it has been disseminated to over 40 countries (Liu et al., 2020). It has demonstrated that interventions that restrict antibiotic application in food animals are closely related to a decrease in the presence of ARB and ARGs (Tang et al., 2017). Secondly, the authorities should publish the guidelines for the prudent application of antibiotics in food animals, strictly limiting the type, usage, amount of antibiotics used, and only allowing their use for therapeutic purposes, to reduce the lowest indispensable level and minimize the development of bacterial resistance (Ungemach et al., 2006). Of note, farmers do not use combinations of antibiotics in the same production cycle, which leads to a higher frequency of multi-drug resistance in pathogenic bacteria than single-drug treatment, especially on broiler farms (Jibril et al., 2021). Additionally, the licensing rules of antibiotics would be made more stringent and the authorities should impose penalties on defaulters (Figure 4; Lee et al., 2013).

**TABLE 4:** Some antibiotics prohibited from use in food animals.

Antibiotics	Countries	Time
All antibiotics as growth promoters	The European Union countries	2006
Lincomycin hydrochloride, virginiamycin, oleandomycin, and procaine penicillin	The United States	2016
Chloramphenicol, clenbuterol, diethylstilbestrol, dimetridazole, ipronidazole/other nitroimidazoles, furazolidone/nitrofurazone, sulfonamide, fluoroquinolones, glycopeptides, phenylbutazone, and cephalosporins	FDA	2017
11 Antibiotics (including bacitracin zinc, flavomycin, virginiamycin, nosiheptide, avilamycin, kitasamycin, oxytetracycin calcium, aureomycin, enlamycin, bacitracin methylene disalicylate, and quinolone premix	China	2020

Generally, concerted efforts are needed to minimize antibiotic resistance in animal farms, especially the development of novel technologies, alternatives, and appropriate use of antibiotics.

## Conclusion

The increasing use of antibiotics in animals over the past century has led to the widespread transmission of ARB and AGRs between animals and humans. Here, we have mainly reviewed the current situation of ARB and ARGs from animal farms, animal manure, and wastewater, as well as potential risks of bacterial resistance in animal farms. We also proposed the development of novel technologies (such as nanotechnology, anaerobic digestion, and biochar composting), alternatives to antibiotics (including herbal plant extracts, probiotics, vaccines, etc.), and antibiotic administration to reduce ARB and ARGs in animal farms, which may help us address the issues of antibiotic resistance.

## Author Contributions

CX helped to investigate, supervise, and wrote the manuscript. LK wrote the manuscript and prepared the figures. HG and XC helped to investigate and review the manuscript. XW conceptualized and wrote the manuscript. All authors read and approved the final manuscript.

## Conflict of Interest

The authors declare that the research was conducted in the absence of any commercial or financial relationships that could be construed as a potential conflict of interest.

## Publisher’s Note

All claims expressed in this article are solely those of the authors and do not necessarily represent those of their affiliated organizations, or those of the publisher, the editors and the reviewers. Any product that may be evaluated in this article, or claim that may be made by its manufacturer, is not guaranteed or endorsed by the publisher.
